# Development of a live attenuated vaccine candidate for equid alphaherpesvirus 1 control: a step towards efficient protection

**DOI:** 10.3389/fimmu.2024.1408510

**Published:** 2024-07-03

**Authors:** Yue Hu, Guiling Wu, Qinrui Jia, Baozhong Zhang, Wencheng Sun, Ruixue Sa, Siyu Zhang, Weifan Cai, Jarhen, Duoliang Ran, Jianhua Liu

**Affiliations:** ^1^ Laboratory of Animal Infectious Disease, College of Veterinary Medicine, Xinjiang Agricultural University, Urumqi, Xinjiang Uygur Autonomous Region, China; ^2^ Chinese Academy of Sciences (CAS) Key Laboratory of Quantitative Engineering Biology, Shenzhen Institute of Synthetic Biology, Shenzhen Institutes of Advanced Technology, Chinese Academy of Sciences, Shenzhen, Guangdong, China; ^3^ Preventive Control Section, Aksu Regional Animal Disease Control and Diagnostic Center, Aksu, Xinjiang Uygur Autonomous Region, China; ^4^ Food, Agricultural and Health Products Division, Centre Testing International Group Co., Ltd., Qingdao, Shandong, China; ^5^ Product Manufacturing Sector, GemPharmatech Co., Ltd., Shanghai, China

**Keywords:** equid alphaherpesvirus 1, live attenuated vaccine, CRISPR-Cas9 genome editing, golden Syrian hamster model, pathogenicity, immune response

## Abstract

Equid alphaherpesvirus 1 (EqAHV1) is a viral pathogen known to cause respiratory disease, neurologic syndromes, and abortion storms in horses. Currently, there are no vaccines that provide complete protection against EqAHV1. Marker vaccines and the differentiation of infected and vaccinated animals (DIVA) strategy are effective for preventing and controlling outbreaks but have not been used for the prevention of EqAHV1 infection. Glycoprotein 2 (gp2), located on the envelope of viruses (EqAHV1), exhibits high antigenicity and functions as a molecular marker for DIVA. In this study, a series of EqAHV1 mutants with deletion of gp2 along with other virulence genes (TK, UL24/TK, gI/gE) were engineered. The mutant viruses were studied *in vitro* and then in an *in vivo* experiment using Golden Syrian hamsters to assess the extent of viral attenuation and the immune response elicited by the mutant viruses in comparison to the wild-type (WT) virus. Compared with the WT strain, the YM2019 Δgp2, ΔTK/gp2, and ΔUL24/TK/gp2 strains exhibited reduced growth in RK-13 cells, while the ΔgI/gE/gp2 strain exhibited significantly impaired proliferation. The YM2019 Δgp2 strain induced clinical signs and mortality in hamsters. In contrast, the YM2019 ΔTK/gp2 and ΔUL24/TK/gp2 variants displayed diminished pathogenicity, causing no observable clinical signs or fatalities. Immunization with nasal vaccines containing YM2019 ΔTK/gp2 and ΔUL24/TK/gp2 elicited a robust immune response in hamsters. In particular, compared with the vaccine containing the ΔTK/gp2 strain, the vaccine containing the ΔUL24/TK/gp2 strain demonstrated enhanced immune protection upon challenge with the WT virus. Furthermore, an ELISA for gp2 was established and refined to accurately differentiate between infected and vaccinated animals. These results confirm that the ΔUL24/TK/gp2 strain is a safe and effective live attenuated vaccine candidate for controlling EqAHV1 infection.

## 1 Introduction

Equid alphaherpesvirus 1 (EqAHV1) is a widespread virus that can cause rhinopneumonia, abortion, and neonatal foal death in equine animals. Certain strains of EqAHV1 can also cause equine herpesvirus myeloencephalopathy (EHM), which poses a significant threat to the equine breeding and racing industries (1–3). The utilization of vaccination in conjunction with management strategies represents a highly effective approach for addressing EqAHV1 infection (4). Modified live or attenuated viral vaccines typically elicit robust humoral and cellular immune responses in vaccinated animals, surpassing the efficacy of killed vaccines (5–7). Nevertheless, existing live vaccines for EqAHV1 do not confer comprehensive immunity against the virus and its neurological complications, leading to persistent outbreaks of EqAHV1-related abortion storms and EHM (8, 9). Therefore, there is a need to develop more effective live attenuated vaccines. To develop a successful marker vaccine candidate and implement the differentiation of infected and vaccinated animals (DIVA) strategy, virulence factors, immune regulators, ideal marker molecules, or genes that are not essential for viral replication are typically deleted (10, 11). Gene 71, which encodes glycoprotein 2 (gp2), controls the recruitment of leukocytes to the equine respiratory tract, allowing the virus to evade the host’s early immune response (12, 13). Furthermore, the gp2 protein is a suitable marker molecule for EqAHV1 due to the enrichment of serine and threonine residues and its high immunogenicity as a glycoprotein (14). An experimental vaccine containing a 71-gene deletion mutant virus was evaluated and found to provide protection against pulmonary disease in mice after challenge with wild-type (wt) EqAHV1 (15). In a previous study, we isolated the EqAHV1 strain YM2019 from the lung tissue of an aborted horse fetus. This strain caused neurological signs in hamsters and horses (16). We constructed a deletion mutant of gp2, strain YM2019 Δgp2, but this strain still caused neurological signs in hamsters. It is necessary to consider the deletion of both gp2 and additional virulence genes to enhance the safety of the live attenuated virus, stimulate the host immune response adequately, and improve overall protection. The genes 37 (UL24), 38 (TK), 73 (gI), and 74 (gE) have been shown to be associated with the virulence of EqAHV1 in natural hosts. The UL24 gene is one of the causative factors of EqAHV1-related lung infection and neurological disease in horses (17, 18); the TK gene is the main virulence factor involved in the pathogenicity of EqAHV1 in the equine nervous system (19, 20); and the gI and gE genes are involved in the spread of the virus via intercellular transmission and chemotactic movement toward the CNS (21, 22). The potential of these genes as targets for gene deletion vaccine candidates has been evaluated. In this study, we constructed three gene-deleted attenuated strains (ΔTK/gp2, ΔUL24/TK/gp2, and ΔgI/gE/gp2) on the basis of the EqAHV1 YM2019 Δgp2 strain using CRISPR/Cas9 technology. We investigated the growth characteristics, virulence, and immunogenicity of the mutant viruses in animal models to determine their suitability as live attenuated vaccine candidates.

## 2 Materials and methods

### 2.1 Virus and cells

EqAHV1 YM2019 was preserved at the China General Microbiological Culture Collection Center. The genome sequence was deposited in GenBank as MT063054. All recombinant strains were constructed from YM2019. Rabbit kidney (RK-13) cells were used throughout the study and were grown in high-glucose Dulbecco’s modified Eagle medium (DMEM) supplemented with 10% fetal bovine serum (FBS) and antibiotics.

### 2.2 Generation of the EqAHV1 YM2019 ΔTK/gp2, ΔUL24/TK/gp2, and ΔgI/gE/gp2 strains

Four homologous donor plasmids were constructed by using two segments flanking the TK, UL24/TK, and gI/gE genes. The fragments of the gene arms were amplified via PCR with corresponding primers. These PCR products were inserted into the pUC19 vector. Then, homologous donor plasmids (HDR-TK, HDR-UL24/TK, and HDR-gI/gE) were obtained. The full-length cDNA encoding enhanced green fluorescence protein (EGFP) and CMV promoter driving EGFP expression was generated via PCR from pEGFP-C plasmid DNA and subsequently cloned and inserted into the HDR-TK, HDR-UL24/TK and HDR-gI/gE vectors. Finally, homologous donor plasmids harboring EGFP (HDR-TK-EGFP, HDR-UL24/TK-EGFP, and HDR-gI/gE-EGFP) were obtained. Guide RNAs targeting the EqAHV1 TK, UL24/TK, gI/gE and EGFP genes were developed using E-CRISP version 5.4 online software (http://www.e-crisp.org/E-CRISP/). The guide RNAs were synthesized, cloned and inserted into the pX330 plasmid (Addgene, 42230). All primers and sgRNAs used in this study are listed in **Supplementary Table 1**. Homologous recombination and CRISPR/Cas9 technology were used simultaneously to construct the gene-deleted viruses (23). RK-13 cells were cotransfected using Lipofectamine 3000 (Invitrogen, USA). In brief, 1 μg of the homologous donor plasmid carrying EGFP, 3 μg of the EqAHV1 YM2019 Δgp2 genome, and 1 μg of the pX330-sgRNA plasmid were cotransfected into RK-13 cells. The cells were cultured for 4–6 days until a large amount of green fluorescence and a corresponding cytopathic effect (CPE) was observed. These cells were harvested by two freeze−thaw cycles, and the recombinant viruses were subjected to five rounds of plaque purification. Plaques with green fluorescence were selected under a fluorescence microscope, amplified, and identified via PCR and DNA sequencing analysis using specific primers (**Supplementary Table 1**). Then, 1 μg of the homologous donor plasmids, 3 μg of the recombinant virus genome, and 1 μg of the pX330-EGFP-sgRNA plasmid were cotransfected into RK-13 cells. Similarly, plaques without green fluorescence were removed, amplified, and identified.

### 2.3 Virus replication kinetics and plaque size determination

Multistep growth curves were generated to evaluate the kinetics of virus replication (24). RK-13 cells were infected with the EqAHV1 YM2019, Δgp2, ΔTK/gp2, ΔUL24/TK/gp2, and ΔgI/gE/gp2 strains at a multiplicity of infection (MOI) of 0.1. The infected cells were collected at 6, 12, 24, 36, 48, 60, and 72 hours post infection (hpi), and the viral titer was determined by calculating the 50% tissue culture infection dose (TCID_50_) at the indicated time points. The replication kinetics curves were generated four times. Plaque sizes were determined at 48 h after inoculation with the virus at an MOI of 0.1. After the cells were incubated with the virus for 1 h, the medium was removed, and 1% low-melting point agarose containing 2% FBS in DMEM was added to form plaques (25). The size of 100 randomly selected plaques for each virus was determined using NIS-Elements Viewer software (Nikon Group). The values were calculated relative to that of EqAHV1 YM2019, which was set at 100%. The average percentages and standard deviations were calculated from three independent experiments.

### 2.4 Animals and experimental design

#### 2.4.1 Infection of Syrian hamsters

Thirty-six female specific pathogen-free (SPF) Syrian hamsters (4 weeks old) were obtained from Charles River Laboratories (CRL). The hamsters were randomly divided into six groups, including five infection groups and a control group (n=6). The infection groups were intranasally inoculated with 0.1 ml of PBS containing 10^8^ TCID_50_ of the viruses. The inoculation dose was selected based on the previously determined 50% lethal concentration (LD_50_) of the WT YM2019 virus. The control group hamsters were inoculated with 0.1 ml of PBS without virus challenge. Body weight was recorded daily, and animals were euthanized if necessary to alleviate suffering. At 8 days postinoculation (dpi), samples were taken from the brain, lungs, and lymph nodes of each hamster in the infection groups for pathologic examination and viral load testing. Histopathological examination was conducted using hematoxylin-eosin (HE) staining (26). Scoring of lung and brain tissues was performed following the principles of histopathologic scoring (27). Lung tissue included five lesion parameters: edema, epithelial thickening, fibrosis, interstitial pneumonia and neutrophilic inflammation. brain tissue included five lesion parameters: nonsuppurative encephalitis, gliosis, neuronal necrosis, neuro-vacuolar degeneration, neutrophilic inflammation. Each parameter was scored according to an ordinal scale as 0 - normal, 1 - mild, 2 - moderate, 3 - severe. The total lung histopathology score and the total brain histopathology score for each hamster were 15 points (**Supplementary Tables 3, 4**). The viral DNA load was determined using primers and a specific probe targeting ORF68 through real-time PCR (28).

#### 2.4.2 Vaccination and challenge of Syrian hamsters

To determine the protective efficacy of YM2019 ΔTK/gp2 and YM2019 ΔUL24/TK/gp2 against lethal challenge with the WT virus, sixty SPF Syrian hamsters were randomly divided into ten groups, including eight vaccinated groups, an unvaccinated group, and a control group (n = 6). The hamsters in the vaccinated groups were intranasally inoculated with 0.1 ml of PBS containing either 10^8^ TCID_50_, 10^7^ TCID_50_, 10^6^ TCID_50_, or 10^5^ TCID_50_ of the YM2019 ΔTK/gp2 or YM2019 ΔUL24/TK/gp2 strain. The control group hamsters were inoculated with 0.1 ml of PBS. At 21 days post-vaccination (dpv), hamsters in the vaccinated groups and control group were challenged via intranasal delivery of 10^8.15^ TCID_50_ wt YM2019. At 14 days post-challenge (dpc), all the surviving hamsters were euthanized and necropsied, and different organ samples were collected for pathologic examination and viral load testing.

### 2.5 Serological test

Serum samples were collected at 0, 7, 14, 21, 28, and 35 dpv. Serum levels of anti-gG and anti-gp2 autoantibodies (IgG) were measured by indirect enzyme-linked immunosorbent assay (I-ELISA) (29). The peak values for IgG responses (anti-gG) were determined by area under the curve (AUC) analyses (30). Serum samples were heat-inactivated at 56°C for 30 min. Fifty microlitres of diluted sera were incubated with 50 μL of WT virus (200 TCID_50_) for 1 h at 37°C. The mixture was added to RK-13 cells in a 96-well plate. The cells were then cultured for 4 days and evaluated for CPEs under a microscope. The titers of the neutralizing antibodies were calculated as the reciprocals of the highest serum dilutions at which no CPE was observed (31).

## 3 Results

### 3.1 *In vitro* characterization of mutant viruses

**Figure 1:**
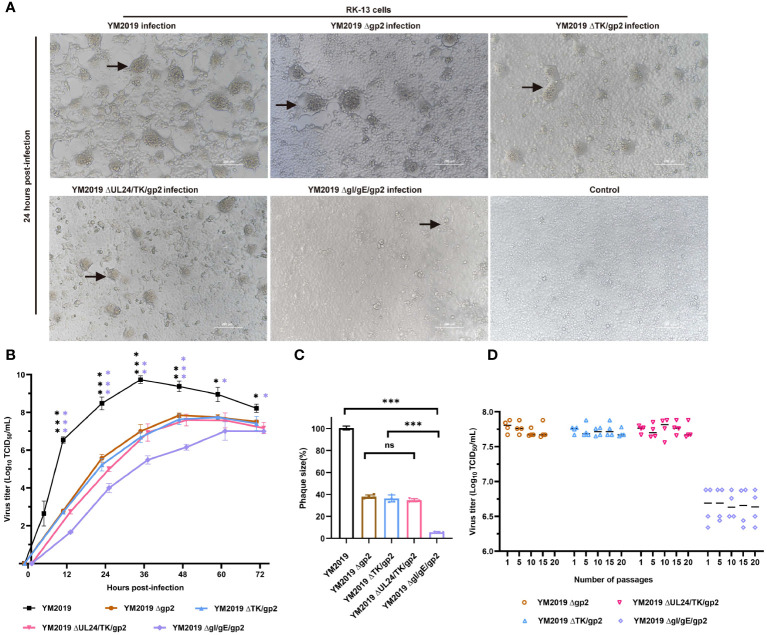
Growth characterization of mutant viruses in RK-13 cells. **(A)** CPE. After 24 h, RK-13 cells were infected with wild-type EqAHV1 YM2019 or the mutants at an MOI of 0.01. The arrows point to syncytia. **(B)** Replication kinetics curves. **(C)** Comparison of plaque sizes. Statistical differences compared with YM2019 (black asterisks) or gE (purple asterisks) were determined by one-way ANOVA. ns (non-significant) P > 0.05, *P < 0.05, **P < 0.01, ***P < 0.001. **(D)** Mutant viruses at passages 1, 5, 10, 15, and 20 were collected for RK-13 cell infection at an MOI of 0.01, and viral titers were determined at 36 (h) P>0.05.

To generate the indicated mutant viruses, we applied CRISPR/Cas9 technology to rapidly knock out the TK, UL24/TK, gI/gE genes. Using the Δgp2 mutant, we constructed a ΔTKΔgp2 double mutant (**Supplementary Figure 1**). Because the UL24 and TK genes are adjacent to the EqAHV1 genome, the UL24 and TK genes were simultaneously knocked out (**Supplementary Figure 2**). Similarly, we simultaneously knocked out the gI gene and gE in the Δgp2 strain and constructed the ΔgI/gEΔgp2 triple mutant (**Supplementary Figure 3**). The deletion of the four target regions was confirmed by PCR and Sanger sequencing, as shown in **Supplementary Material**. CPEs, including syncytium formation, rounding, and vacuolization, were observed in RK-13 cells after infection with the WT or deletion mutant strains for 24 h (**Figure 1A**). However, the gI/gE/gp2 mutant exerted a weaker CPE than wild-type EqAHV1 YM2019 and other mutant strains at a multiplicity of infection (MOI) of 0.1.

A comparison of the *in vitro* growth kinetics of these virus mutants in RK-13 cells with those of the wild-type (WT) virus showed that replication was significantly attenuated (**Figure 1B**). In particular, compared with the wild-type (WT) virus, the ΔgI/gEΔgp2 mutant showed an approximately 2.7-log reduction in the peak viral titer. Plaque assays revealed that the plaques created by Δgp2, ΔTK/gp2, and ΔUL24/TK/gp2 were smaller than those produced by the WT virus (~60% less), and ΔgI/gEΔgp2 produced plaques that were significantly smaller than those produced by the WT virus (~94% less) (**Figure 1C**). These findings demonstrate that ΔgI/gEΔgp2 exhibited severe growth deficiency, indicating that viral replication was severely impeded by simultaneous deletion of gI/gE and gp2. The stability of mutant growth was assessed *in vitro* by determining the viral titers of the four mutant viruses in RK-13 cells after 1, 5, 10, 15 and 20 passages. As shown in **Figure 1D**, there were no significant differences in the titers of the deletion mutant viruses at different passages (P > 0.05). This indicates that the viral titers of the deletion mutant viruses remained stable after serial passaging of the RK-13 cells.

### 3.2 Pathogenicity of EqAHV1 YM2019, Δgp2, ΔTK/gp2, and ΔUL24/TK/gp2 in Syrian hamsters

**Figure 2:**
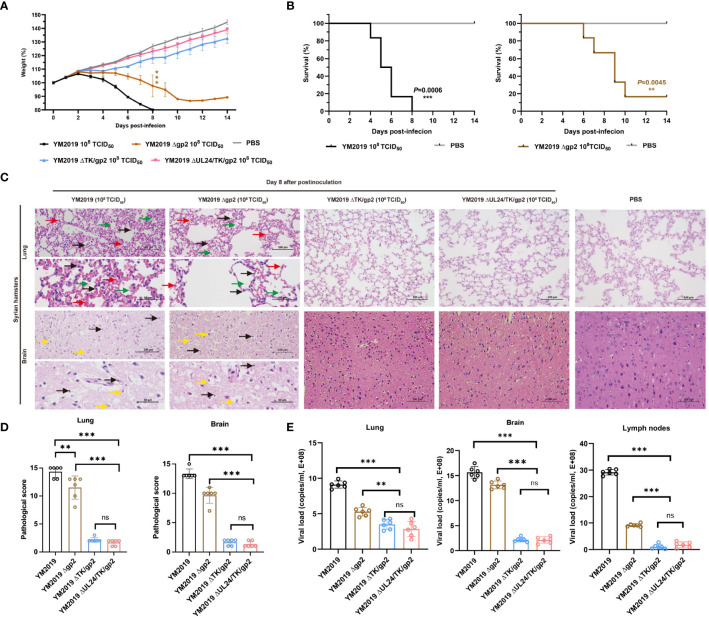
Pathogenicity of mutant viruses in Syrian hamsters. **(A)** Body weight loss and **(B)** the survival rate were monitored until day 14. **(C)** Pathological lesions in the lung and brain tissues were detected using HE staining. The inflammatory exudates in the alveolar spaces are indicated by green arrows, alveolar hemorrhage by red arrows, neuronal swelling by yellow arrows, and inflammatory cell infiltration by black arrows. **(D)** Lesion scoring of lung and brain tissue in different groups. **(E)** Viral load in lung and brain tissue and lymph nodes as determined by RT−qPCR. ns P > 0.05, **P < 0.01, ***P < 0.001.

To assess the virulence of the mutant viruses, we inoculated groups of six animals intranasally with 10^8^ TCID_50_ of either the mutant viruses or the WT strain. At 3 dpi, hamsters in the WT and Δgp2 infection groups exhibited clinical signs, including circling, involuntary shaking of the head and forelimbs, cowering, shivering, shortness of breath, and vomiting, and experienced significant weight loss compared to those in the control group (P < 0.001) (**Figure 2A**). All hamsters infected with the WT strain died by 8 dpi, whereas a single hamster infected with the Δgp2 strain survived until 14 dpi. The survival rate of hamsters in the Δgp2 infection group was 16.67%. (**Figure 2B**). Severe pathological damage was observed in the lung and brain tissues of hamsters in both the Δgp2- and WT-infected groups. Significant increase of the alveolar septum thickness was observed, with widespread thickening of the alveolar septa due to a preponderance of macrophages, as well as a few neutrophils and lymphocytes, as shown in **Figure 2C**. Inflammatory cells extensively infiltrated the brain tissue, while the neurons displayed edema with an inflated, vacuolized morphology (**Figure 2C**).

Compared with that of the WT strain, the virulence of the mutant strains ΔTK/gp2 and ΔUL24/TK/gp2 was decreased. The ΔTK/gp2 or ΔUL24/TK/gp2 infection groups did not exhibit any significant animal mortality, clinical signs, abnormal body weight changes, or pathological damage to the lung or brain tissues during the fourteen-day experimental period (**Figure 2C**). The average lesion scores of lung and brain tissue were less than 5 points in ΔTK/gp2, and ΔUL24/TK/gp2-infected groups, significant difference from WT (P < 0.001) (**Figure 2D**). The results demonstrated that the DNA loads in the lung, brain, and lymph nodes were significantly lower in hamsters inoculated with ΔTK/gp2 or ΔUL24/TK/gp2 than in those inoculated with the WT strain (P < 0.001) (**Figure 2E**) after 14 dpi. This finding suggests that Δgp2 causes disease in hamsters after intranasal infection, while ΔTK/gp2 and ΔUL24/TK/gp2 are not virulent in hamsters.

### 3.3 Protection of immunized hamsters against EqAHV1 challenge

**Figure 3:**
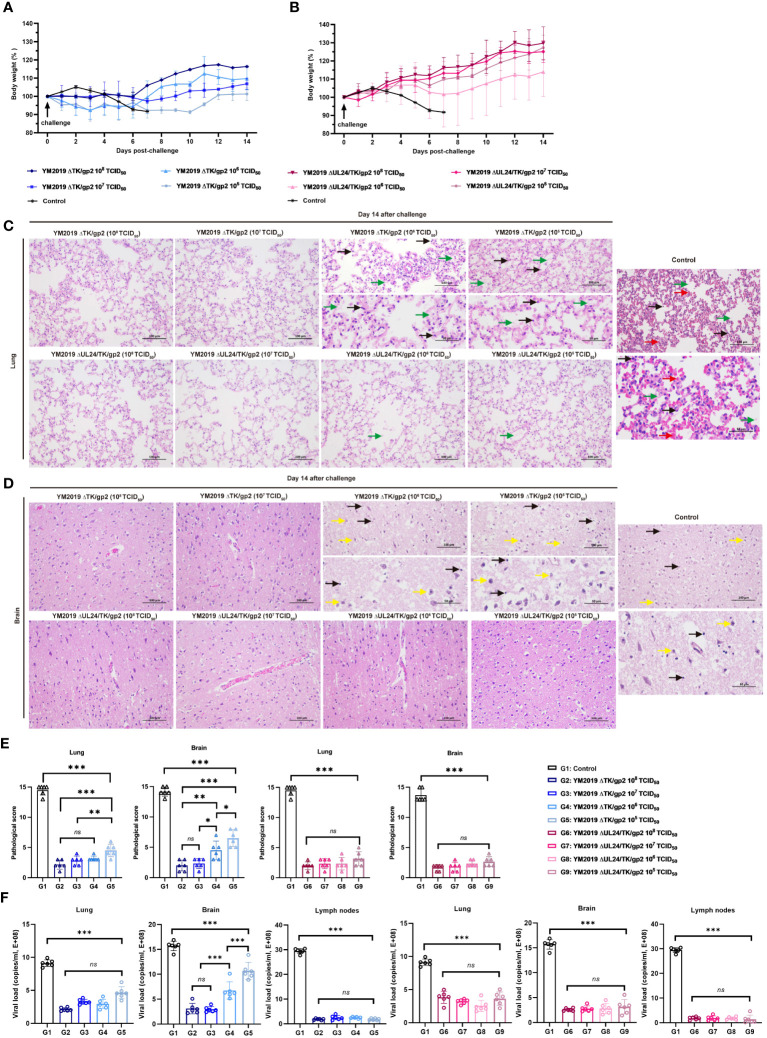
Protection of ΔTKΔgp2 and ΔUL24/TKΔgp2-immunized hamsters against wild-type strain challenge. Body weights of **(A)** ΔTKΔgp2-immunized hamsters and **(B)** ΔUL24/TKΔgp2-immunized hamsters until 14 dpc. Histopathological lesions in the **(C)** lung and **(D)** brain tissues of the hamsters at 14 dpc. HE on all hamsters were performed at time of death of the control group. The inflammatory exudates in the alveolar spaces are indicated by green arrows, alveolar hemorrhage by red arrows, neuronal swelling by yellow arrows, and inflammatory cell infiltration by black arrows. **(E)** Lesion scoring of lung and brain tissue in different groups. **(F)** Viral load in lung and brain tissue and lymph nodes. ns P > 0.05, *P < 0.05, **P < 0.01, ***P < 0.001.

We assessed the level of protection against challenge with the WT strain following immunization with four different doses of ΔTK/gp2 and ΔUL24/TK/gp2. Notably, the body weights of the hamsters in all the ΔUL24/TK/gp2 immunization groups remained stable (**Figure 3B**), and the hamsters did not exhibit any clinical signs during the 14-day challenge period. These results provide strong evidence for the efficacy of the vaccine. All hamsters in the ΔTK/gp2 immunization groups experienced weight loss to varying degrees (**Figure 3A**). Notably, the two hamsters in the 10^5^ TCID_50_/dose ΔTK/gp2-immunized group exhibited clinical signs within 6 dpv, such as dullness, depressed behavior, serous nasal discharge, and dyspnea, but none of the hamsters died.

Lung and brain tissue pathology was examined through anatomical observation and HE staining after a 14-day challenge. The hamsters in the ΔTK/gp2-immunized group exhibited notable pathological alterations in their lung and brain tissues, with the exception of those receiving the 10^8^ and 10^7^ TCID_50_ dose (**Figures 3C–E**). Conversely, hamsters immunized with varying doses of ΔUL24/TK/gp2 did not display any discernible abnormalities in their lung and brain tissues, except for mild inflammation observed in the 10^6^ TCID_50_/dose and 10^5^ TCID_50_/dose groups. The average lesion scores were below 5 points in all ΔUL24/TK/gp2-immunization groups, demonstrating a significant difference from the unimmunized group (P < 0.001) (**Figure 3E**). This confirms the effectiveness of ΔUL24/TK/gp2 immunization in preventing abnormalities in lung and brain tissue. Additionally, the DNA loads in the lung, brain, and lymph nodes were significantly lower in all hamsters immunized with ΔUL24/TK/gp2 than in those in the unimmunized group (P < 0.001) (**Figure 3F**). The results unequivocally demonstrate that immunization with ΔUL24/TK/gp2 generated a robust immune response that effectively protected the hamsters against subsequent WT virus challenge even at doses that were not protective for YM2019 ΔTK/gp2.

### 3.4 Antibody production in hamsters

**Figure 4:**
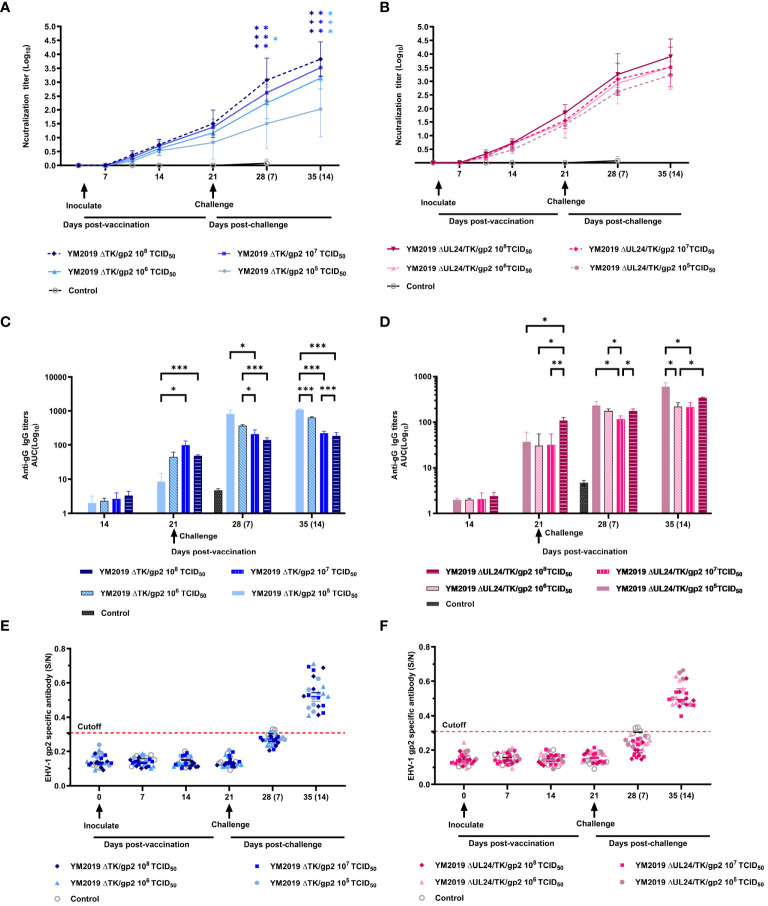
Serological assays. The levels of neutralizing antibodies of **(A)** ΔTKΔgp2-immunized and **(B)** ΔUL24/TKΔgp2-immunized hamsters were tested in RK-13 cells. ELISA was utilized to measure the anti-EqAHV1 gG antibody levels of **(C)** ΔTKΔgp2-immunized and **(D)** ΔUL24/TKΔgp2-immunized hamsters, and the anti-EqAHV1 gp2 antibody levels of **(E)** ΔTKΔgp2-immunized and **(F)** ΔUL24/TKΔgp2-immunized hamsters. The dashed line indicates the cutoff value. The arrow indicates the challenge time. S/N ratio, sample OD450 divided by the negative control. S/N > 0.55, positive; S/N < 0.55, negative. *P < 0.05, **P < 0.01, ***P < 0.001.

An *in vitro* neutralization assay was conducted to assess the neutralizing ability of anti-EqAHV1 antibodies induced by ΔUL24/TK/gp2 and ΔTK/gp2. The production of EqAHV1-specific neutralizing antibodies was induced in all vaccinated hamsters after 21 days post-vaccination, and the levels increased rapidly after the challenge. Regarding ΔTK/gp2 immunization, the group that received the 10^8^ TCID_50_ dose had significantly greater neutralization titers at 14 dpc (P<0.01) than did the group that received the 10^5^ TCID_50_ dose (**Figure 4A**). However, the differences among the ΔUL24/TK/gp2-immunized groups were not significant (P > 0.05) (**Figure 4B**).

ELISA was used to detect antibody responses to two EqAHV1 antigens (gG and gp2) before and five weeks after immunization (weekly testing). At 14 dpv, gG-specific antibodies were detected in all vaccine groups, albeit at low levels. By 21 dpv, antibody levels had increased in all vaccine groups (**Figures 4C, D**). Following challenge with the EqAHV1 YM2019 strain, a significant difference was observed between the group that received 10^5^ TCID_50_ of ΔTK/gp2 and the other three groups at 14 dpc (P < 0.001) (**Figure 4C**). All groups produced gp2-specific antibodies after the challenge, which were detected at 14 dpc (**Figures 4E, F**), despite none of the groups producing gp2-specific antibodies before the challenge. The results indicate that gp2 can be a reliable marker for distinguishing between infected and vaccinated animals using ELISA.

## 4 Discussion

EqAHV1 has been a significant cause of economic losses in the horse industry since its discovery in 1947 (32). Research on EqAHV1 vaccines began in the 1990s, with only gE-deleted vaccines undergoing early clinical trials (33–35). However, surveys have shown that gE-deleted vaccines can only provide partial protection against EqAHV1 infection (36). Immunized horses still experience viremia, and some develop fever during the period of viremia (37). To identify a vaccine candidate with sufficiently attenuated and reduced immunosuppressive effects, we constructed four strains of EqAHV1: the Δgp2, ΔTK/gp2, ΔUL24/TK/gp2, and ΔgI/gEΔgp2 gE strains. Our findings indicate that EqAHV1 ΔUL24/TK/gp2 had a greater protective effect on Syrian hamsters than the other strains. In the challenge protection experiment, all hamsters in the control group died, while none of the hamsters immunized with EqAHV1 ΔUL24/TK/gp2 died or exhibited clinical signs. Therefore, the challenge protection rate was 100% for the immunized hamsters. While there are differences in the pathogenesis of EqAHV1 between Syrian hamsters and horses, hamsters are highly susceptible to EqAHV1 and can develop severe neurological and respiratory diseases. Thus, they have been used to study the pathogenicity and immunoprotective efficacy of attenuated strains (38–40). This study demonstrated that the ΔUL24/TK/gp2 strain elicited a robust protective anti-EqAHV1 immune response in hamsters. This suggests that ΔUL24/TK/gp2 could be a potential vaccine candidate for managing EqAHV1, and clinical trials in horses are necessary.

EqAHV1 is propagated primarily using RK-13 cells, which are the most commonly used cell type for this purpose (41, 42). The study revealed that compared with wt EqAHV1, an EqAHV1 gp2 single-gene deletion mutant exhibited decreased growth in RK-13 cells, as indicated by a markedly reduced plaque area and virus titers. This highlights the importance of the gp2 gene in EqAHV1 propagation using RK-13 cells. Compared with Δgp2, ΔgI/gE/gp2 had cumulative effects and caused a significant decrease in viral proliferation efficiency. The loss of syncytial lesions resulted in fewer indistinct edges, decreased areas, and altered CPE. This suggests that the ΔgI/gE/gp2 mutant virus is less capable of engaging in cell-to-cell spread in RK-13 cell (43), and due to its limited proliferative capacity, the strain was not used as a vaccine candidate in this study.

A previous study demonstrated that the YM2019 strain is closely related to the Ab4 strain and can cause the same clinical signs in both hamster models and horses (16). Marshall et al. (15) reported that intranasal inoculation of BALB/c mice with the Ab4 gp2 strain resulted in significant reductions in clinical and pathological manifestations, particularly in terms of respiratory signs and pulmonary pathology. Inoculation also triggered protective immunity against WT EqAHV1. In contrast, the YM2019 gp2 strain still resulted in an 83% mortality rate in Syrian hamsters at high doses (10^8^ TCID_50_/mL). This variation may be attributed to the use of different animal models. Nonetheless, further research is required to determine the pathogenicity of the gp2 deletion strain in horses.

The production of high levels of antibodies by the body to combat viral infections for an extended period after vaccination is crucial (44, 45). The neutralizing antibody assay demonstrated that both mutant viruses induced some neutralizing antibodies after immunization of hamster rats, with antibody levels increasing from day 7 to 21. At 21 days post-immunization, the level of neutralizing antibodies produced in response to ΔUL24/TK/gp2 (10^5^ TCID_50_/mL) was significantly greater than that produced in response to ΔTK/gp2 (10^5^ TCID_50_/mL) (P = 0.043). Correspondingly, none of the animals exhibited clinical signs after receiving any dose of ΔUL24/TK/gp2, whereas hamsters in the low-dose (10^5^ TCID_50_/mL) ΔTK/gp2 group displayed clinical signs after challenge. Furthermore, histological observations of lung and brain tissue revealed that, compared with ΔTK/gp2, ΔUL24/TK/gp2 provided superior protection following both high- and low-dose immunization. This complete protection can be attributed to the strong neutralizing antibody response induced by ΔUL24/TK/gp2 vaccination. UL24, a highly conserved core protein of herpesvirus, has been demonstrated to act on multiple immune signaling pathways in herpes simplex virus type 1(HSV-1) and pseudorabies virus (PRV), thereby participating in immune escape from the host antiviral response (46). However, the role of UL24 in immune escape of EqAHV1 has not yet been established. In this paper, the UL24 gene deletion mutant may have elicited more active stimulation of the adaptive immune response than the EqAHV1 ΔTK/gp2 strain. Further research is required to elucidate the underlying mechanisms involved.

A high-quality vaccine strain should be cost-effective and safe, provide strong immune protection, and allow for easy differential diagnosis (47). In this study, the proliferation, safety, and immunogenicity of the EqAHV1 YM2019 ΔUL24/TK/gp2 strain were evaluated *in vitro*. The results showed that the strain proliferated well in RK-13 cells, was safe, and had good immune effects in Syrian hamsters. In addition, as expected, no anti-gp2 antibody was detected in any hamster before challenge, and the gp2 protein could serve as a serological marker protein. These data suggest that ΔUL24/TK/gp2 may be a promising live attenuated vaccine candidate for controlling EqAHV1 infection and may provide new ideas for vaccine development.

## Data availability statement

The datasets presented in this study can be found in online repositories. The names of the repository/repositories and accession number(s) can be found in the article/**Supplementary Material**.

## Ethics statement

The animal study was approved by The Ethics Committee for Animal Experiments at the Xinjiang Agricultural University. The study was conducted in accordance with the local legislation and institutional requirements.

## Author contributions

YH: Methodology, Writing – original draft, Writing – review & editing. GW: Writing – original draft, Methodology. QJ: Writing – original draft, Investigation. WS: Software, Writing – original draft. RS: Writing – original draft. SZ: Writing – original draft. BZ: Writing – review & editing. WC: Writing – original draft. J: Resources, Writing – original draft. DR: Funding acquisition, Writing – review & editing. JL: Funding acquisition, Writing – review & editing.
