# Supplementary material for: Development of a live attenuated vaccine candidate for equid alphaherpesvirus 1 control: a step towards efficient protection
Source: Front Immunol. 2024 Jul 3;15:1408510. doi: 10.3389/fimmu.2024.1408510 (PMC11252532; doi:10.3389/fimmu.2024.1408510)
Supplement: Supplementary file 1 [file DataSheet_1.pdf]

Supplementary Material

1 Supplementary Figures and Tables

1.1 Supplementary Figures

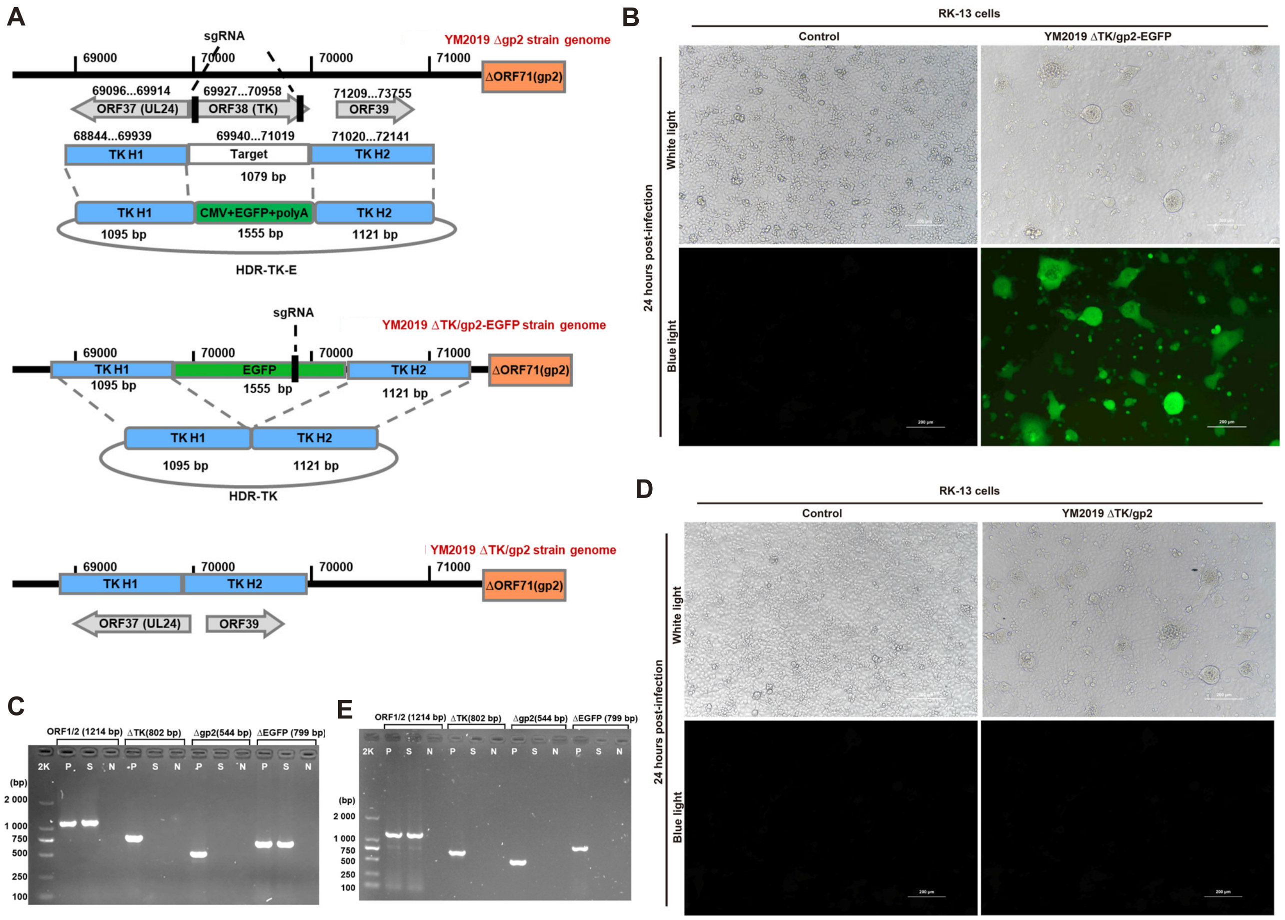

Supplementary Figure 1. Construction and identification of a TK/gp2 gene deletion mutant virus (EqAHV1 YM2019ΔTK/gp2). (A) The EGFP cassette was inserted using the Cas9 system based on the Δgp2 mutant virus, and the TK gene was replaced with the EGFP cassette to create the ΔTK/gp2-EGFP mutant. (B) The CPE and green fluorescence were observed under a fluorescence microscope. (C) The deletion of the TK gene and insertion of the EGFP gene were confirmed through PCR. (D) Under a fluorescence microscope, a CPE and no green fluorescence was observed. (E) PCR confirmed the deletion of the gp2, TK, and EGFP genes.

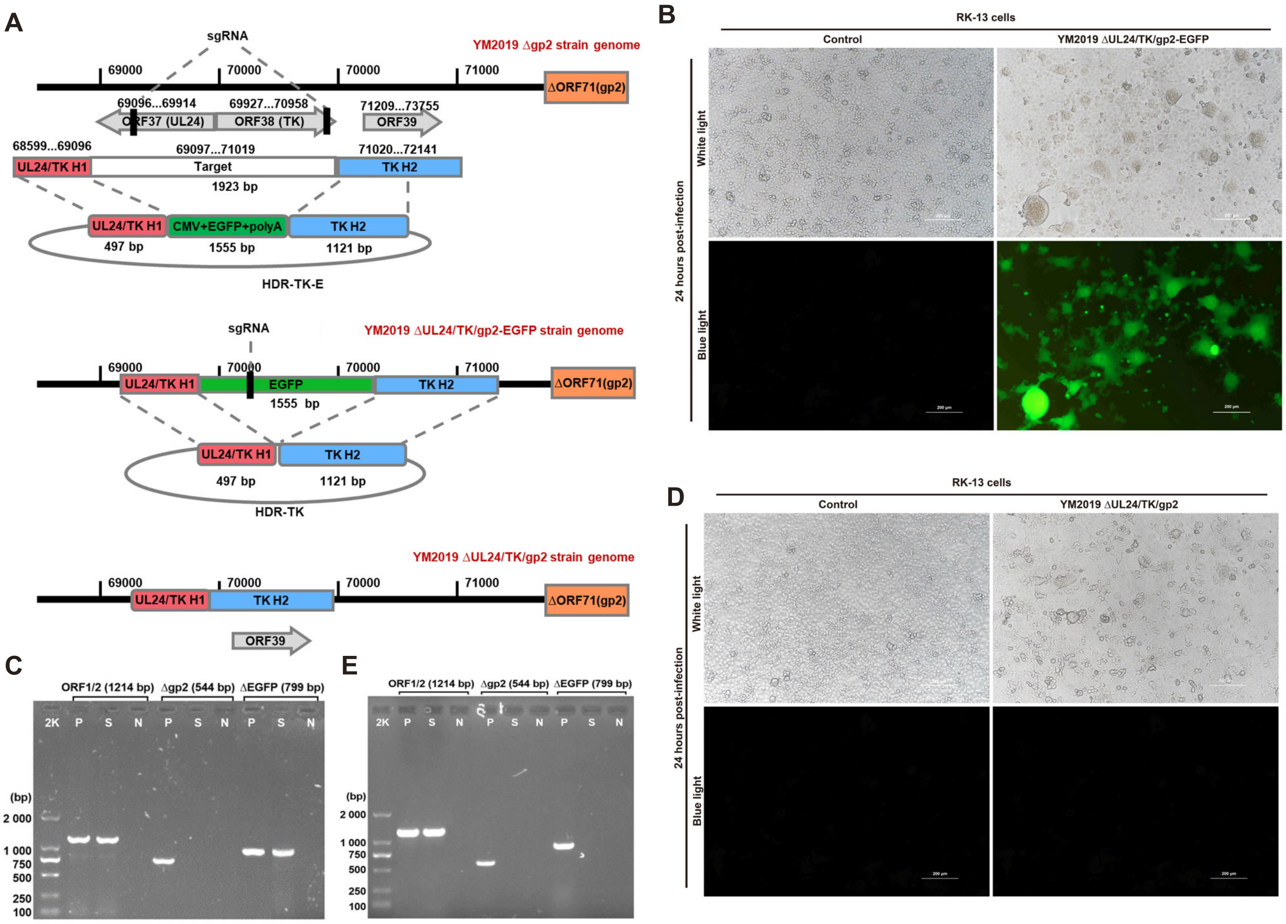

Supplementary Figure 2. Construction and identification of UL24/TK/gp2 genes deletion mutant virus (EqAHV1 YM2019ΔUL24/TK/gp2). (A) The EGFP cassette was inserted using the Cas9 system based on the Δgp2 mutant virus, and the UL24/TK genes was replaced with the EGFP cassette to create the ΔUL24/TK/gp2-EGFP mutant. (B) The CPE and green fluorescence were observed under a fluorescence microscope. (C) The deletion of the UL24 and TK genes and insertion of the EGFP gene were confirmed through PCR. (D) Under a fluorescence microscope, a CPE and no green fluorescence was observed. (E) PCR confirmed the deletion of the UL24, TK, gp2, and EGFP genes.

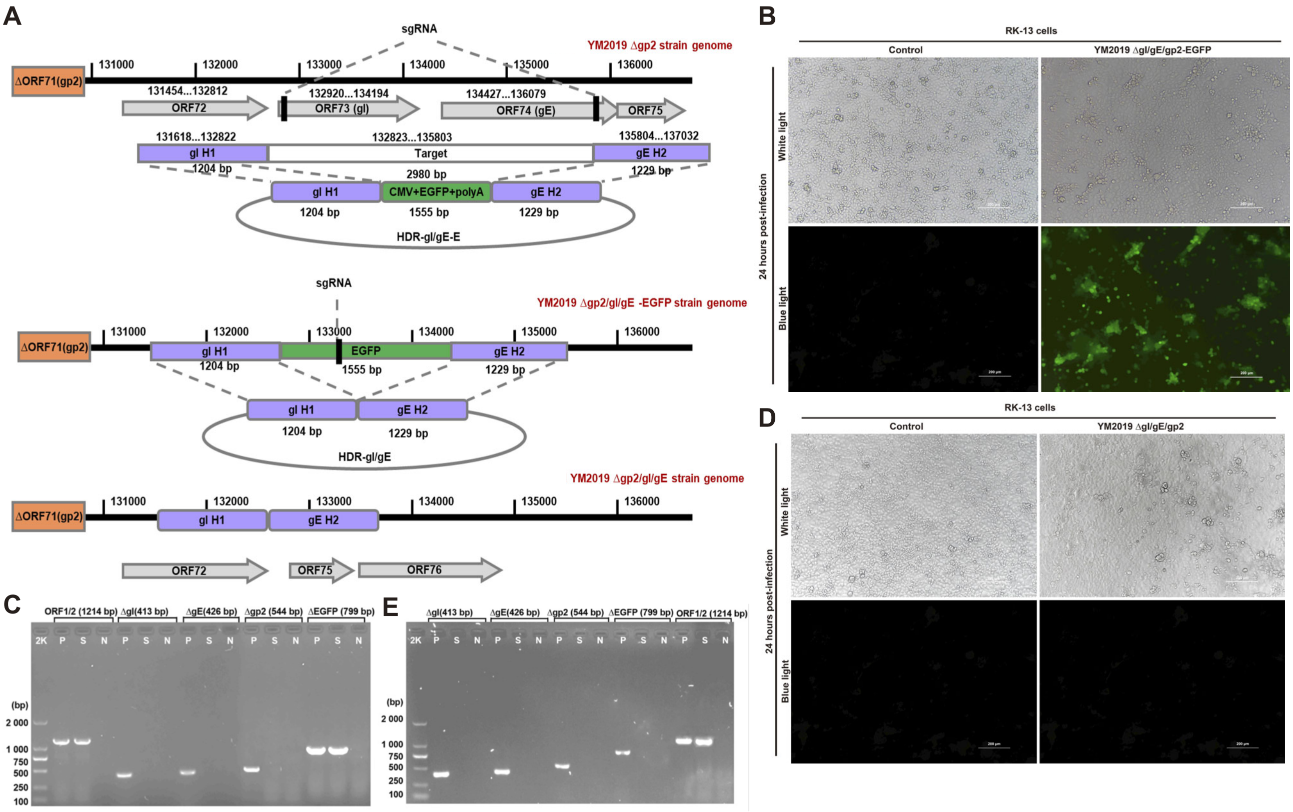

Supplementary Figure 3. Construction and identification of gI/gE/gp2 genes deletion mutant virus (EqAHV1 YM2019ΔgI/gE/gp2). (A) The EGFP cassette was inserted using the Cas9 system based on the Δgp2 mutant virus, and the gI/gE genes was replaced with the EGFP cassette to create the ΔgI/gE/gp2-EGFP mutant. (B) The CPE and green fluorescence were observed under a fluorescence microscope. (C) The deletion of the gI and gE genes and insertion of the EGFP gene were confirmed through PCR. (D) Under a fluorescence microscope, a CPE and no green fluorescence was observed. (E) PCR confirmed the deletion of the gp2, gI, gE, and EGFP genes.

1.2 Supplementary Tables

Supplementary Table 1. Primers used in this study.

Primer name	Sequence (5′-3′)	Purpose
gI/gEH1-F	CCCAAGCTTGCTTATGATGGATGGACGTTTGGT	Upstream homologous arm of gI and ge genes
gI/gEH1-R	CGGGATCCGCAACACTGTTTACGGAAGCTGG	
gI/gEH2-F	GGGGTACCGAGGTCTTGGTGTTTGAGCGC	Downstream homologous arm of gI and ge gene
gI/gEH2-R	CCGGAATTCGATCCCCATTCTCAGAAGCAGC	
TKH1-F	CCCAAGCTTGTGTTGGAGCTGACGGTGAAT	Upstream homologous arm of TK gene
TKH1-R	CGGGATCCCGCGAGCCGCCATTGATTT	
TKH2-F	GGGGTACCATCCCTCATACCGCCTCGTGT	Downstream homologous arm of TK gene
TKH2-R	CCGGAATTCCCTGGAGATCCTGCTCATTCC	
UL24H2-F	CCCAAGCTTGTGTTGGAGCTGACGGTGAAT	Downstream homologous arm of UL24 and TK gene
UL24H2-R	CGGGATCCCCTAAATCGACAAGGAGGCGGTG	
eGFP-F	CGGGATCCTAGTTATTAATAGTAATCAATTACG	eGFP expression cassette
eGFP-R	GGGGTACCATGCAGTGAAAAAAATGCT	
ΔTK-F	AACTCTATACTTTCCTGAGCCTATG	Verification of TK gene deletion
ΔTK-R	AGACTCCACGGTTGCAGC	
ΔgP2-F	CAGCCTCGACTACCTCTGCTAC	Verification of gp2 gene deletion
ΔgP2-R	GAAGGAGTCAAACGGTCTGGGT	
ΔeGFP-F	ATGGTGAGCAAGGGCGAGGA	Verification of eGFP gene deletion
ΔeGFP-R	TTATCTAGATCCGGTGGATCCCGG	
ΔUL24-F	GCTAGTACGATCTCACCGCTT	Verification of UL24 gene deletion
ΔUL24-R	CCACAACCGCTTCTATAACGC	
ΔgI-F	AACCACCGAAACTGAATCC	Verification of gI gene deletion
ΔgI-R	CACCGACAACCACCAACA	
ΔgE-F	CTTGGGCTATGATCGACGGAAGG	Verification of gE gene deletion
ΔgE-R	GTTGTTTGGGCGGCTTCTTGAC	
ORF1-F	GCCATGAGACCCGAGGG	Detection of viral genomes by ORF1 gene.
ORF1-R	GTTGAACCACGTCCCAGAC	
TK-sgRNA1-F	CACCGTACCTTCCGGGGAAGCTCGA	sgRNA targeting gp2 gene
TK-sgRNA1-R	AAACTCGAGCTTCCCCGGAAGGTAC	
TK-sgRNA2-F	CACCGCTCGGCGTTGAAGGTGCGCG	sgRNA targeting TK gene
TK-sgRNA2-R	AAACCGCGCACCTTCAACGCCGAGC	
UL24-sgRNA1-F	CACCGTATTCCGCCAAACGTGATA	sgRNA targeting UL24 gene
UL24-sgRNA1-R	AAACTATCACGTTTGGCGGAATAC	
gI-	CACCGGTGTTGCGTAACCTGCTGGG	sgRNA targeting gI gene

sgRNA1-F		
gI-sgRNA1-R	AAACCCCAGCAGGTTACGCAACACC	
gE-sgRNA2-F	CACCGTCGTCCGAGGGGTCGTTGCT	sgRNA targeting gE gene
gE-sgRNA2-R	AAACAGCAACGACCCCTCGGACGAC	
eGFP-sgRNA-F	CACCGGCGAGGGCGATGCCACCTA	sgRNA targeting eGFP gene
eGFP-sgRNA-R	AAACTAGGTGGCATCGCCCTCGCC	

Supplementary Table 2. Virus strains and plasmids used in this study.

**Strain Number**	**Genotype/Phenotype**	**Source**
YM2019	Wild type EqAHV1	China General Microbiological Culture Collection Center
YM2019 Δgp2	EqAHV1 gp2 gene-deleted virus	This study
YM2019 ΔTKΔgp2	EqAHV1 TK/gp2 dual gene-deleted virus	This study
YM2019 ΔUL24/TKΔgp2	EqAHV1 UL24/TK/gp2 triple gene-deleted virus	This study
YM2019 Δgp2ΔgI/gE	EqAHV1 gp2/gI/gE triple gene-deleted virus	This study
HDR-gp2-E	pUC-19-gp2HDR-eGFP	This study
HDR-gp2	pUC-19-gp2HDR	This study
HDR-TK-E	pUC-19-TKHDR-eGFP	This study
HDR-TK	pUC-19-TKHDR	This study
HDR-UL24/TK-E	pUC-19-UL24/TKHDR-eGFP	This study
HDR-UL24/TK	pUC-19-UL24/TKHDR-eGFP	This study
HDR-gI/gE -E	pUC-19-gI/gE HDR-eGFP	This study
HDR-gI/gE	pUC-19-gI/gEHDR	This study
gp2-sgRNA1	pX330-gp2-sgRNA1	This study
gp2-sgRNA2	pX330-gp2-sgRNA2	This study
TK-sgRNA1	pX330-TK-sgRNA1	This study
TK-sgRNA2	pX330-TK-sgRNA1	This study
UL24/TK-sgRNA1	pX330-UL24/TK-sgRNA1	This study
gI/gE-sgRNA1	pX330-gI/gE-sgRNA1	This study
gI/gE-sgRNA2	pX330-gI/gE-sgRNA2	This study
eGFP- sgRNA1	pX330-eGFP-sgRNA1	This study

Supplementary Table 3. Histopathologic grading of lung tissues in EqAHV1

Histopathologic changes	Histopathologic grading			
	Normal	fatal infection	moderate infection	mild infection
Edema	0	3	2	1
Epithelial thickening	0	3	2	1
Fibrosis	0	2	1	1
Interstitial pneumonia	0	2	2	1
Neutrophilic inflammation	0	3	2	1
Total histological score	0/15	13/15	9/15	5/15

Supplementary Table 4. Histopathologic grading of brain tissues in EqAHV1

Histopathologic changes	Histopathologic grading			
	Normal	fatal infection	moderate infection	mild infection
Nonsuppurative encephalitis	0	3	2	1
Gliosis	0	3	2	1
Neuronal necrosis	0	2	2	1
Neuro-vacuolar degeneration	0	2	1	1
Neutrophilic inflammation	0	3	2	1
Total histological score	0/15	13/15	9/15	5/15
